# Supplementary material for: Non-Viral Systems Based on PAMAM-Calix-Dendrimers for Regulatory siRNA Delivery into Cancer Cells
Source: Int J Mol Sci. 2024 Nov 24;25(23):12614. doi: 10.3390/ijms252312614 (PMC11641217; doi:10.3390/ijms252312614)
Supplement: Supplementary file 1 [file ijms-25-12614-s001.zip › ijms-3308747-supplementary.pdf]

Electronic Supplementary Information

For

Non-viral systems based on PAMAM-calix-dendrimers for regulatory siRNA delivery into cancer cells

Pavel Padnya,[1] Igor Shiabiev,[1] Dmitry Pysin,[1] Tatiana Gerasimova,[2] Bahdan Ranishenka,[3] Alesia Stanavaya,[3] Viktar Abashkin,[3] Dzmitry Shcharbin,[3] Xiangyang Shi, [4,5] Mingwu Shen,[4] Anastasia Nazarova,[1,*] Ivan Stoikov[1,*]

[1] A.M. Butlerov Chemical Institute, Kazan Federal University, 18 Kremlevskaya St., 420008 Kazan, Russia;

[2] Arbuzov Institute of Organic and Physical Chemistry, FRC Kazan Scientific Center, Russian Academy of Sciences, 8 Arbuzov Street, Kazan 420088, Russian Federation;

[3] Institute of Biophysics and Cell Engineering of NASB, 27 Akademicheskaya St., 220072 Minsk, Belarus;

[4] State Key Laboratory for Modification of Chemical Fibers and Polymer Materials, Shanghai Engineering Research Center of Nano-Biomaterials and Regenerative Medicine, College of Biological Science and Medical Engineering, Donghua University, Shanghai 201620, China;

[5] CQM—Centro de Química da Madeira, Universidade da Madeira, Campus Universitário da Penteada, 9020-105 Funchal, Portugal.

* Correspondence: anas7tasia@gmail.com, ivan.stoikov@mail.ru; Tel.: +7-843-233-7241 (I.S.)

Electronic Supplementary Information (14 pages)

Table of Contents

1. NMR spectra of **PAMAM-calix-dendrimers** S3
2. Quantum-chemical calculations data S5
3. DLS data S6
4. Raw data of gel electrophoresis S9
5. SiRNA release from the complexes with **PAMAM-calix-dendrimers** . . S10
6. Temporal stability of the complexes S10
7. Evaluation of siRNA stability in the complexes with **PAMAM-calix-dendrimers** S11
8. Inhibition of PBMC viability by **PAMAM-calix-dendrimers** S12
9. Cellular uptake of PAMAM dendrimer/siRNA complexes S12
10. Fluorescent images of the internalized **PAMAM-calix-dendrimer**/siRNA complexes S13
11. Dose-response curves of HeLa cells with PAMAM dendrimers . . . S14

1. NMR spectra of PAMAM-calix-dendrimers

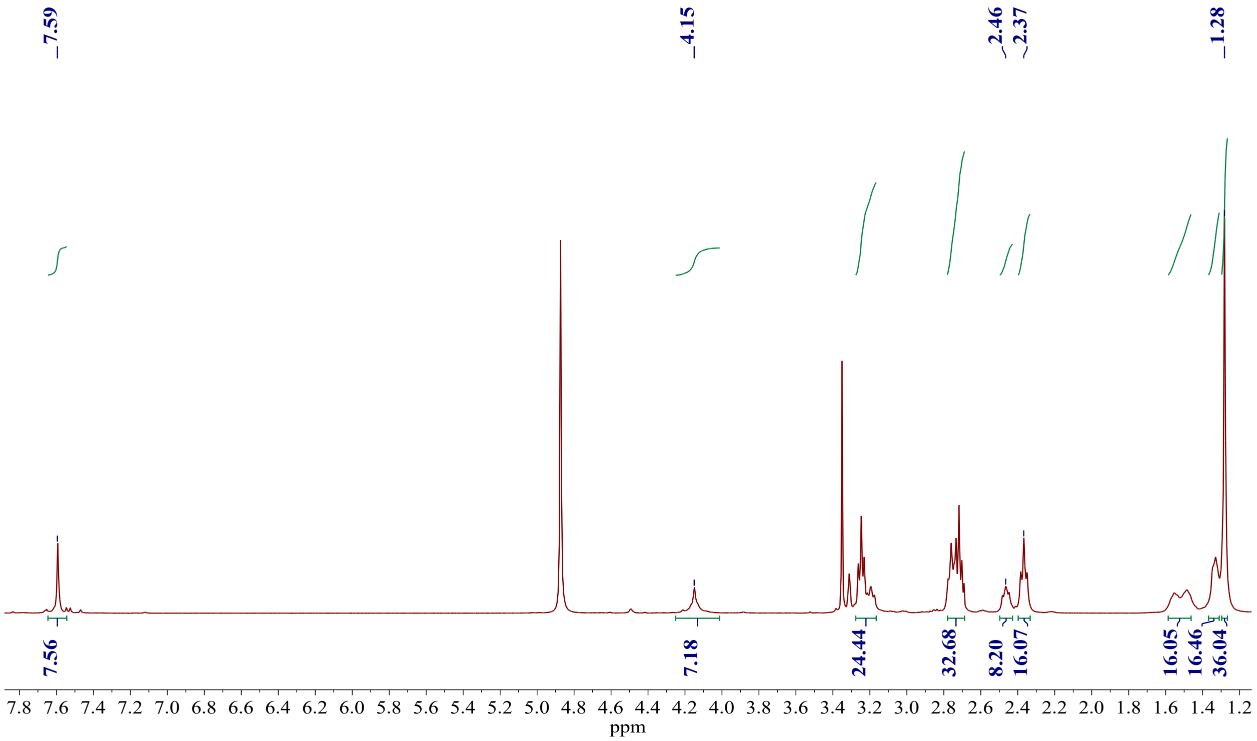

Figure S1. ^{1}H NMR spectrum of **G1-alt**, CD_3OD, 298 K, 400 MHz.

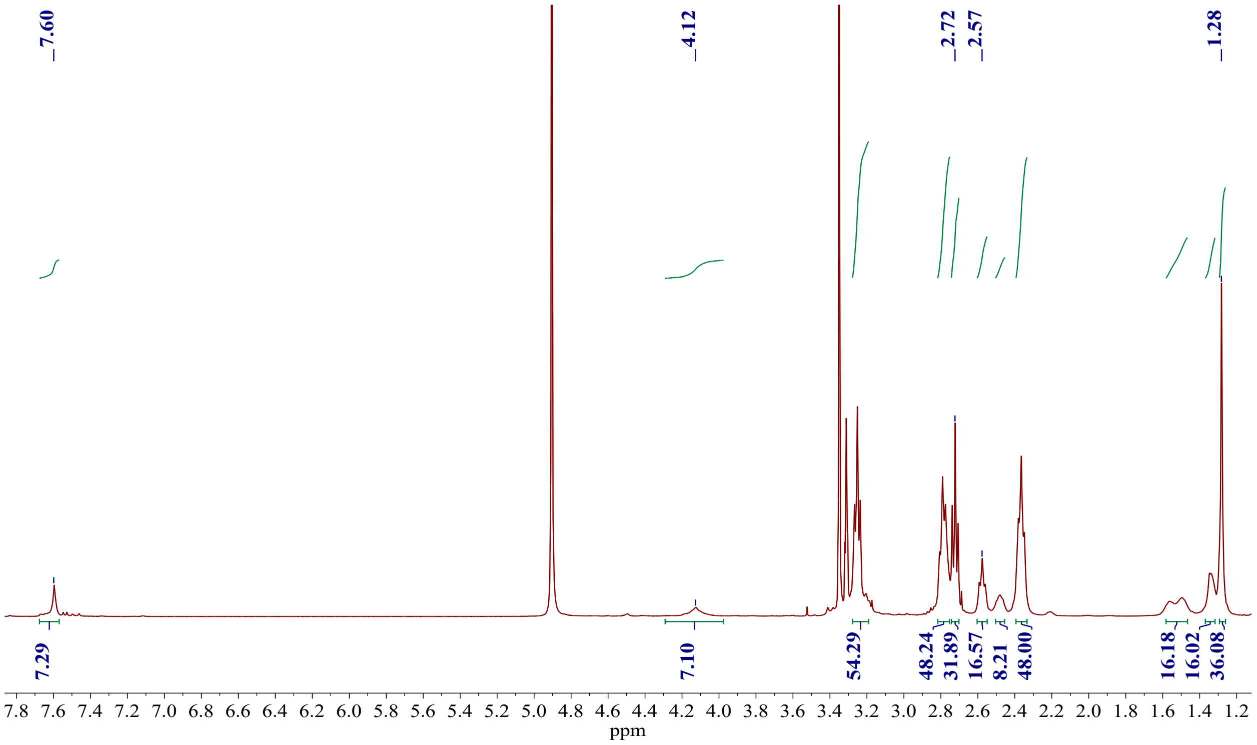

Figure S2. ^{1}H NMR spectrum of **G2-alt**, CD_3OD, 298 K, 400 MHz.

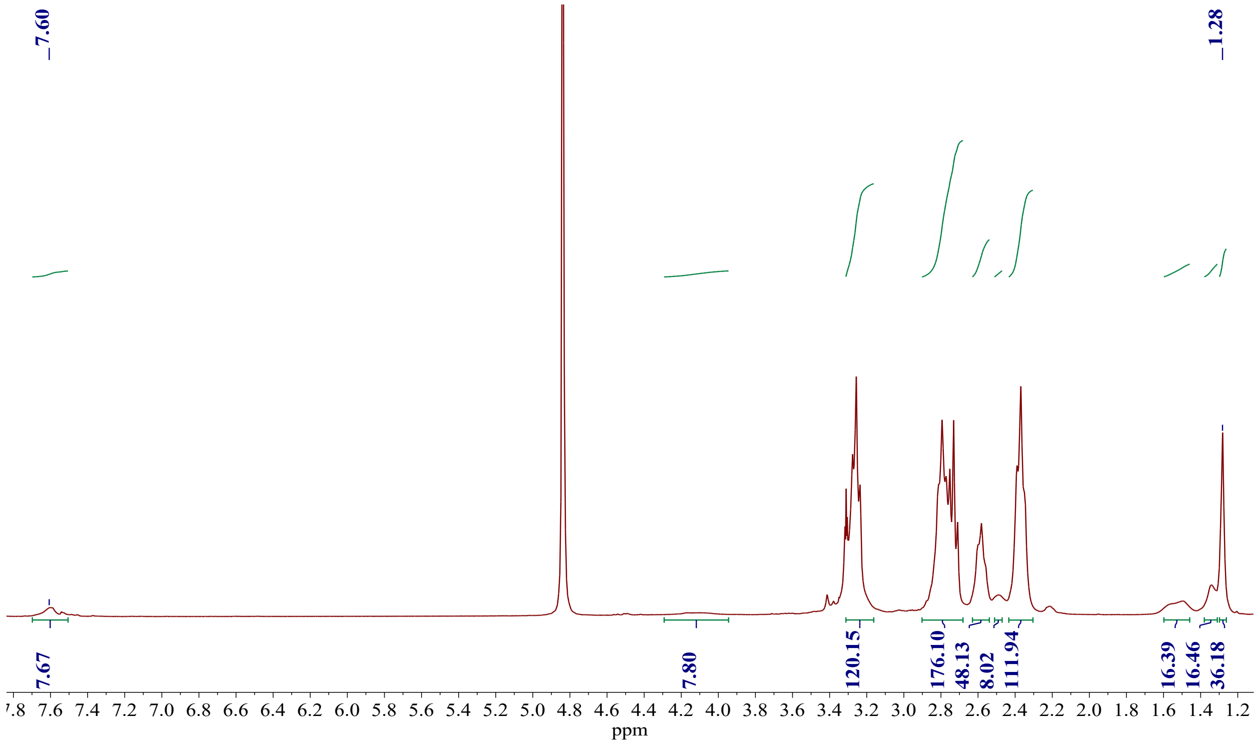

Figure S3. ^{1}H NMR spectrum of **G2-alt**, CD_3OD, 298 K, 400 MHz.

2. Quantum-chemical calculations data

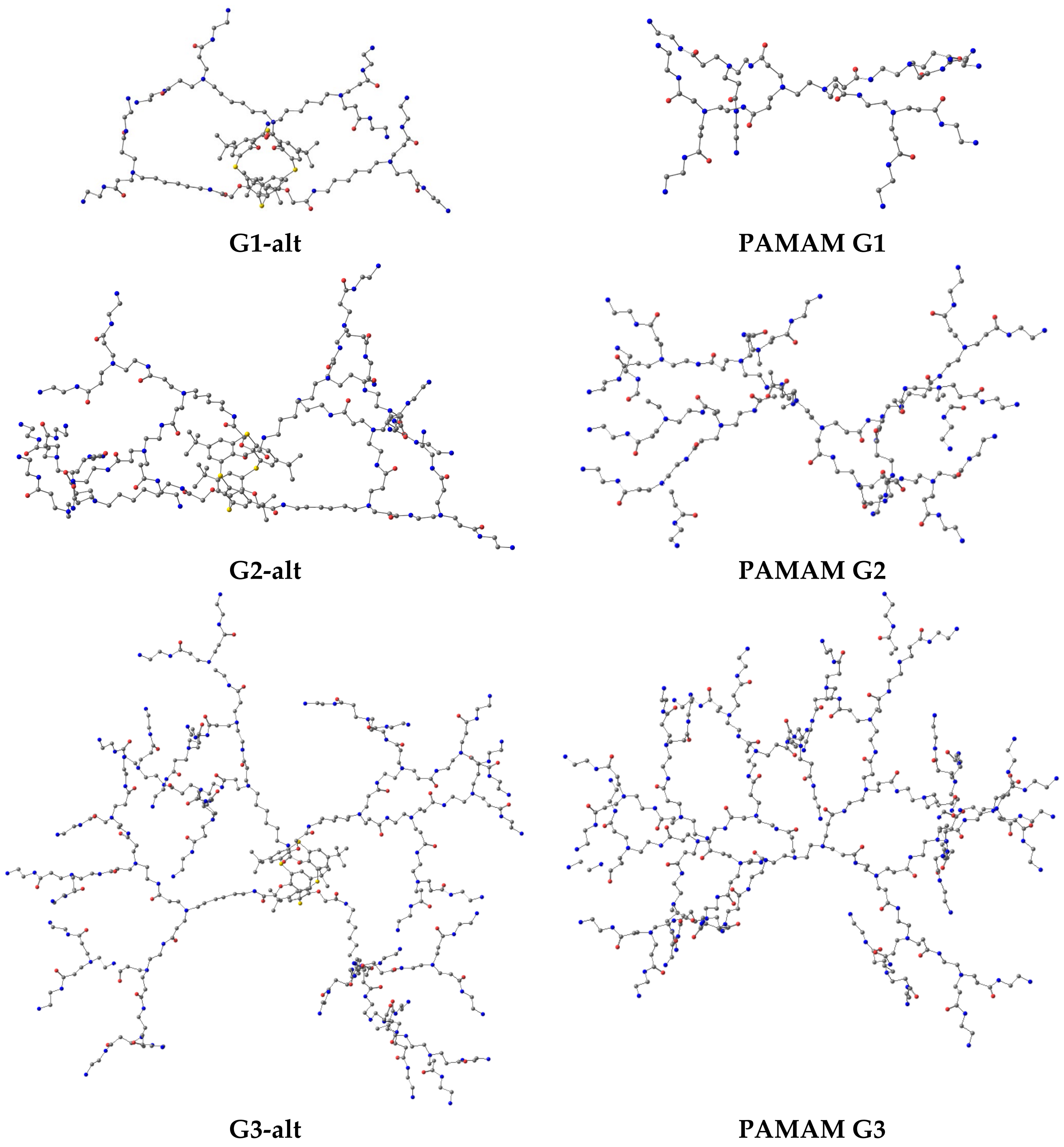

Figure S4. Optimized structures of G1–G3 of **PAMAM-calix-dendrimers** and classical PAMAM dendrimers.

Table S1. Calculated molar volume and xyz "box" data for the dendrimers.

Dendrimer	Molar volume (bohr^3/mol)	Molar volume (cm^3/mol)	x, Å	y (Å)	z (Å)	Volume of xyz "box" (Å^3)
**G1-alt**	35482	3166	41	22	14	12628
**G2-alt**	77422	6909	51	32	25	40800
**G3-alt**	140993	12582	62	52	58	186992
**PAMAM G1**	26482	2363	33	30	14	13860
**PAMAM G2**	56712	5061	46	31	25	35650
**PAMAM G3**	121741	10864	52	57	52	154128

3. DLS data

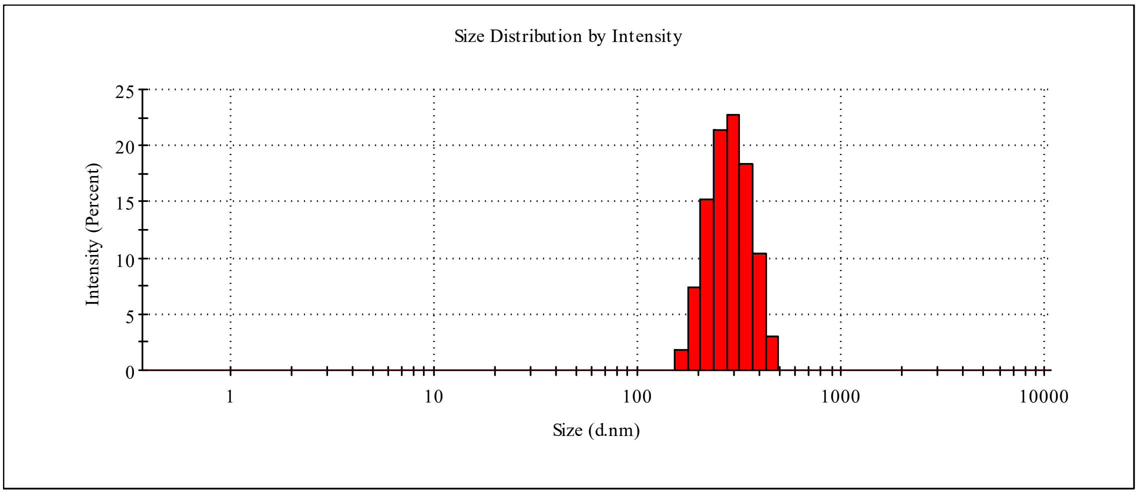

Figure S5. Size distribution of particles by intensity for **G1-alt** (1 μM) in PBS (37 °C).

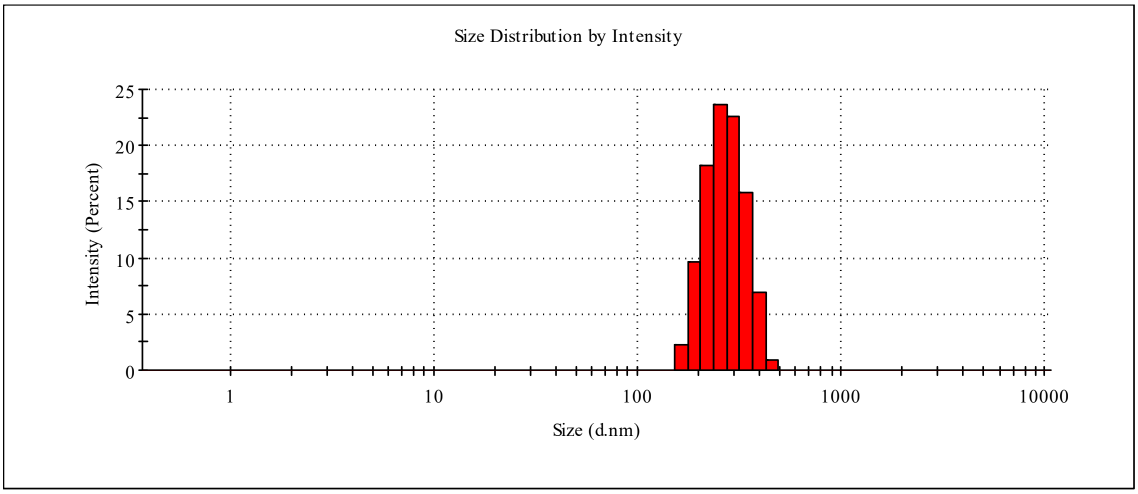

Figure S6. Size distribution of particles by intensity for **G1-alt** (10 μM) in PBS (37 °C).

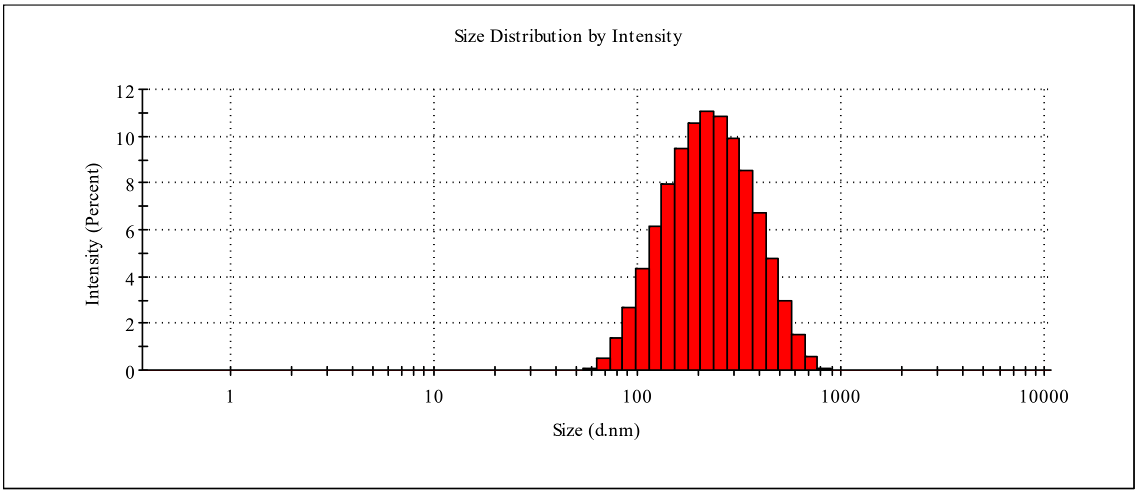

Figure S7. Size distribution of particles by intensity for **G1-alt** (100 μM) in PBS (37 °C).

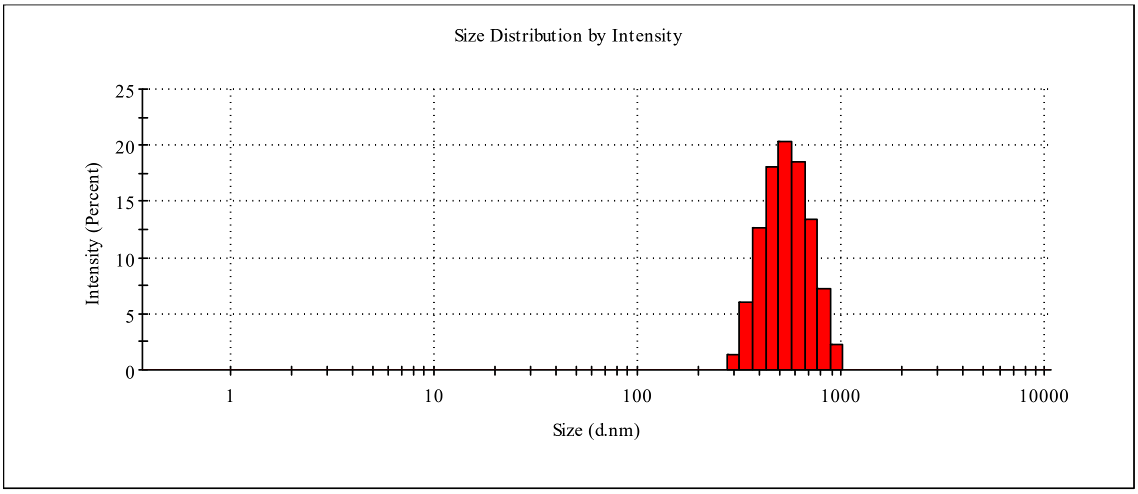

Figure S8. Size distribution of particles by intensity for **G2-alt** (1 μM) in PBS (37 °C).

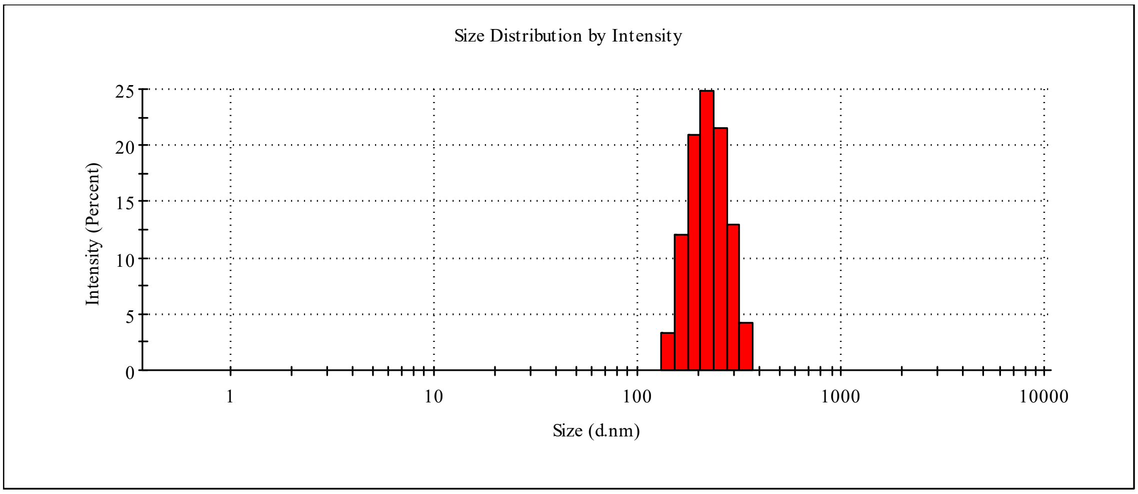

Figure S9. Size distribution of particles by intensity for **G2-alt** (10 μM) in PBS (37 °C).

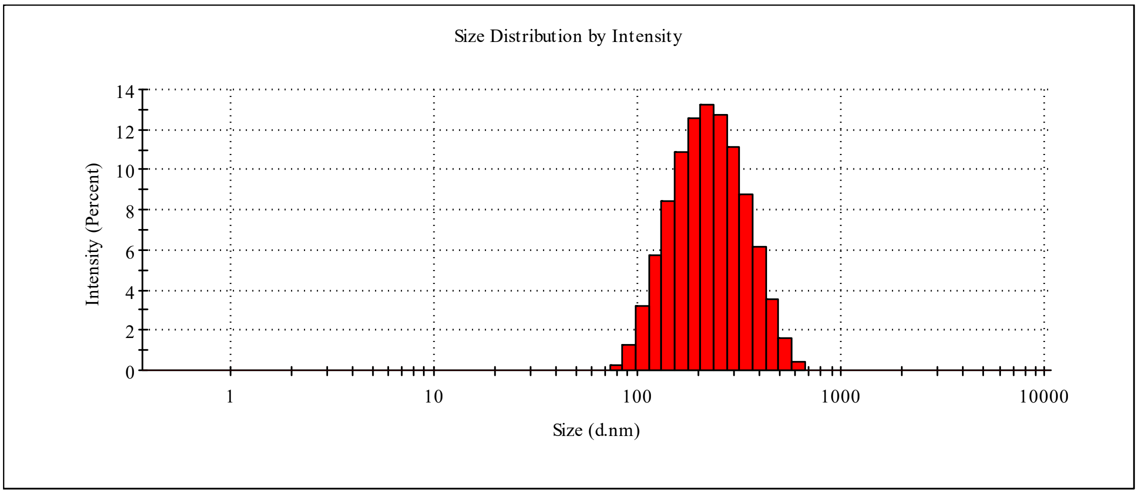

Figure S10. Size distribution of particles by intensity for **G2-alt** (100 μM) in PBS (37 °C).

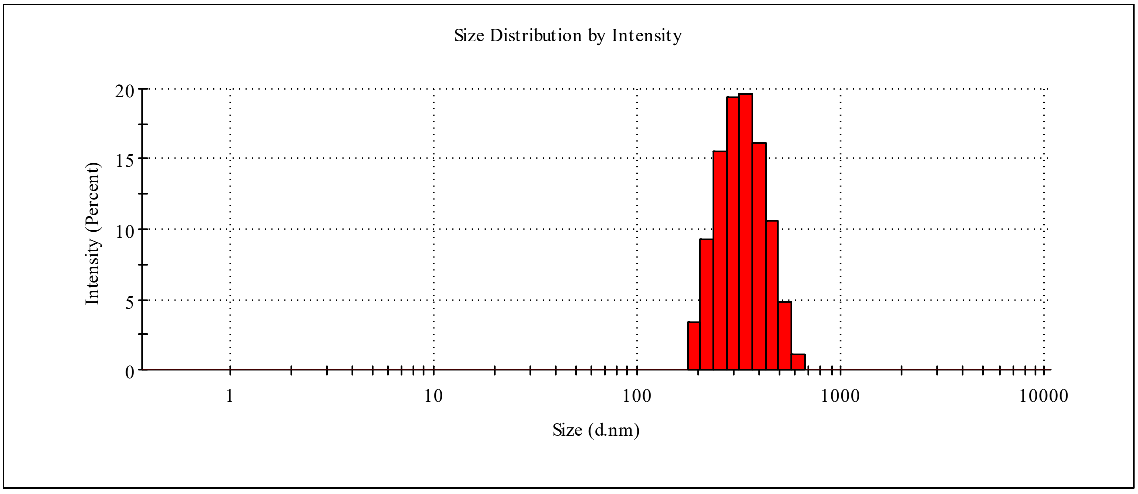

Figure S11. Size distribution of particles by intensity for **G3-alt** (1 μM) in PBS (37 °C).

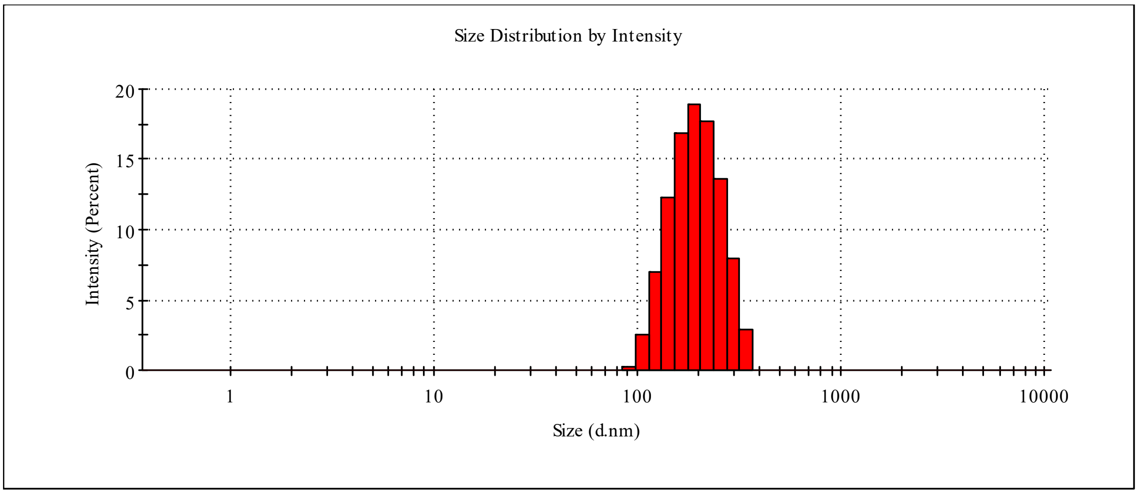

Figure S12. Size distribution of particles by intensity for **G3-alt** (10 μM) in PBS (37 °C).

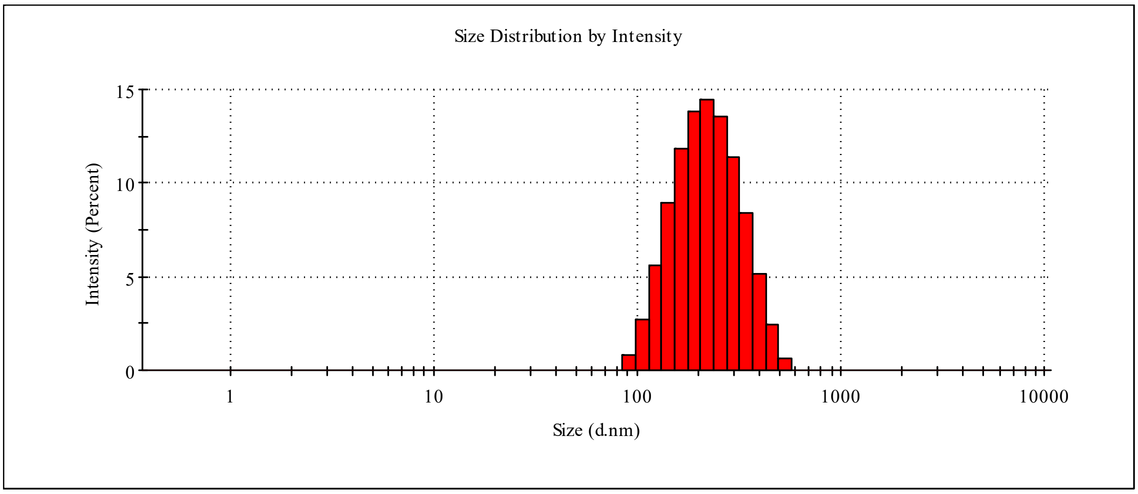

Figure S13. Size distribution of particles by intensity for **G3-alt** (100 μM) in PBS (37 °C).

4. Raw data of gel electrophoresis

(a)

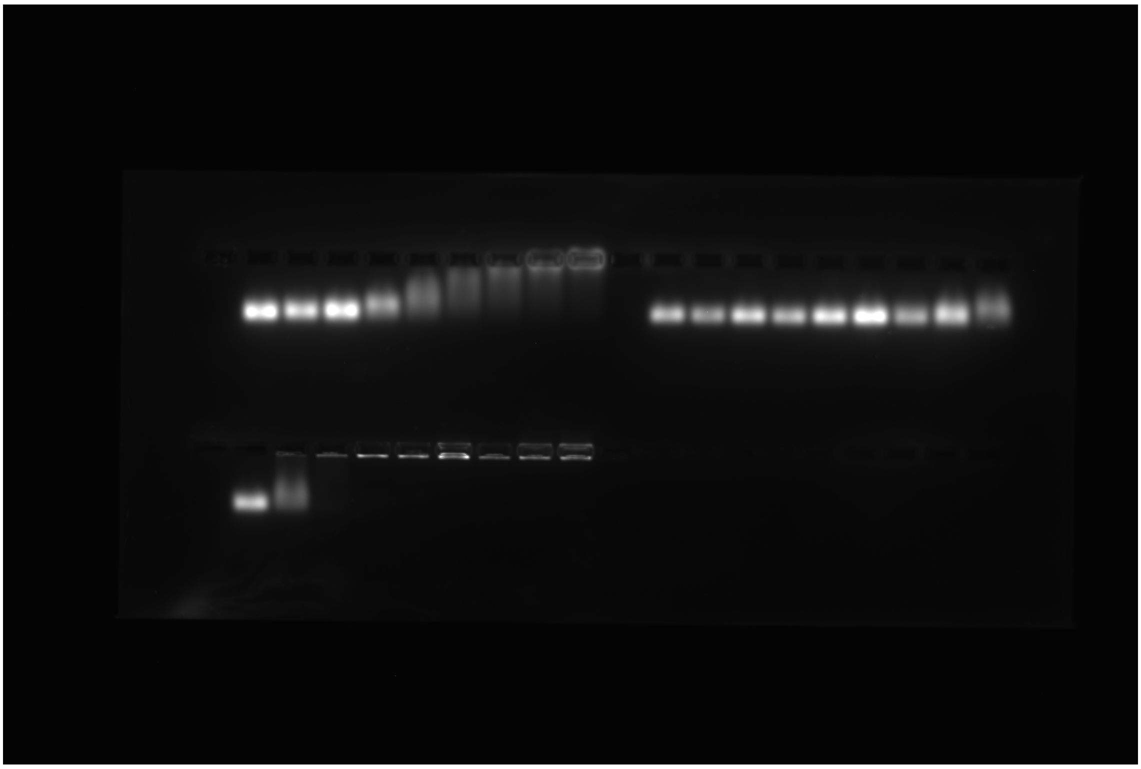

(b)

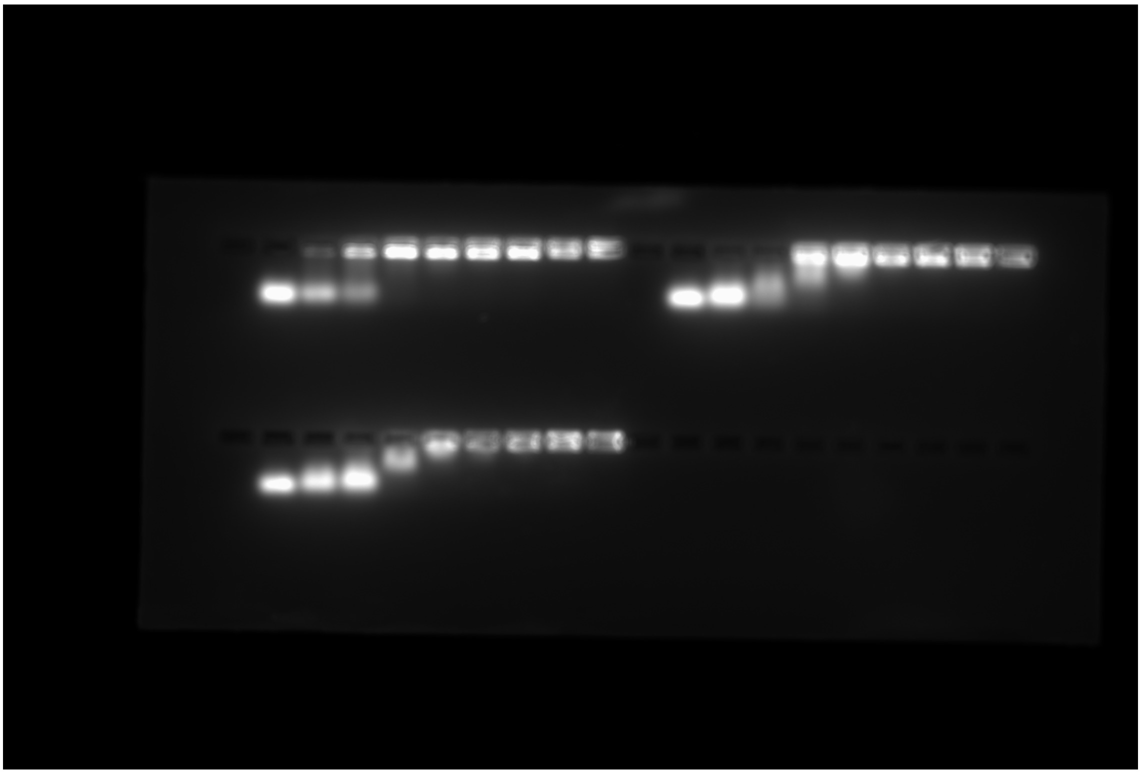

Figure S14. Raw data of gel electrophoresis of the complexes of siRNA with PAMAM dendrimers (a) or **PAMAM-calix-dendrimers** (b) at the varying N:P ratios. Concentration of siRNA (siBCL-2) = 1.5 μM; incubation time, 15 min. "Ctrl" is non-targeted siRNA control well for signal level comparison.

5. SiRNA release from the complexes with PAMAM-calix-dendrimers

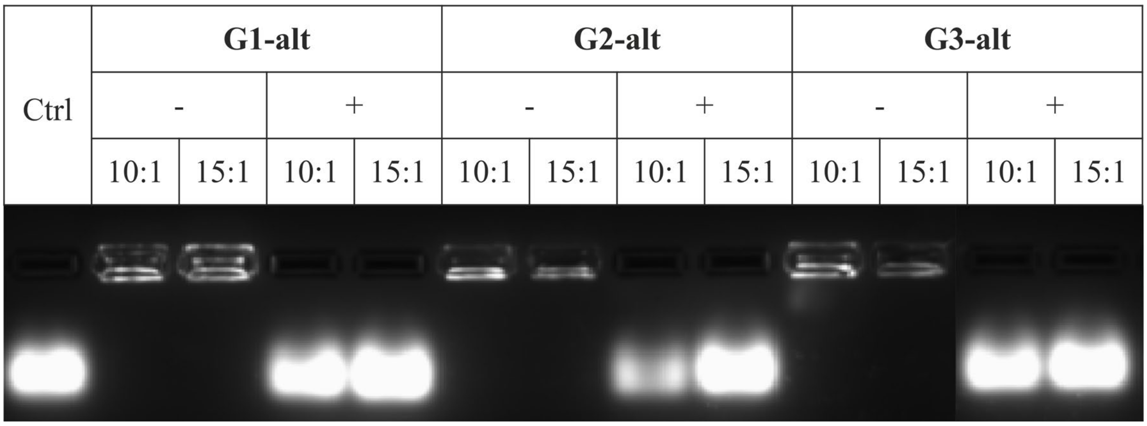

Figure S15. SiRNA release from the complexes with **PAMAM-calix-dendrimers** in (+) the presence and (–) in the absence of heparin. siBCL-2 concentration = 1.5 μM; TAE buffer, electrophoresis parameters: 30 min, 40 V.

6. Temporal stability of the complexes

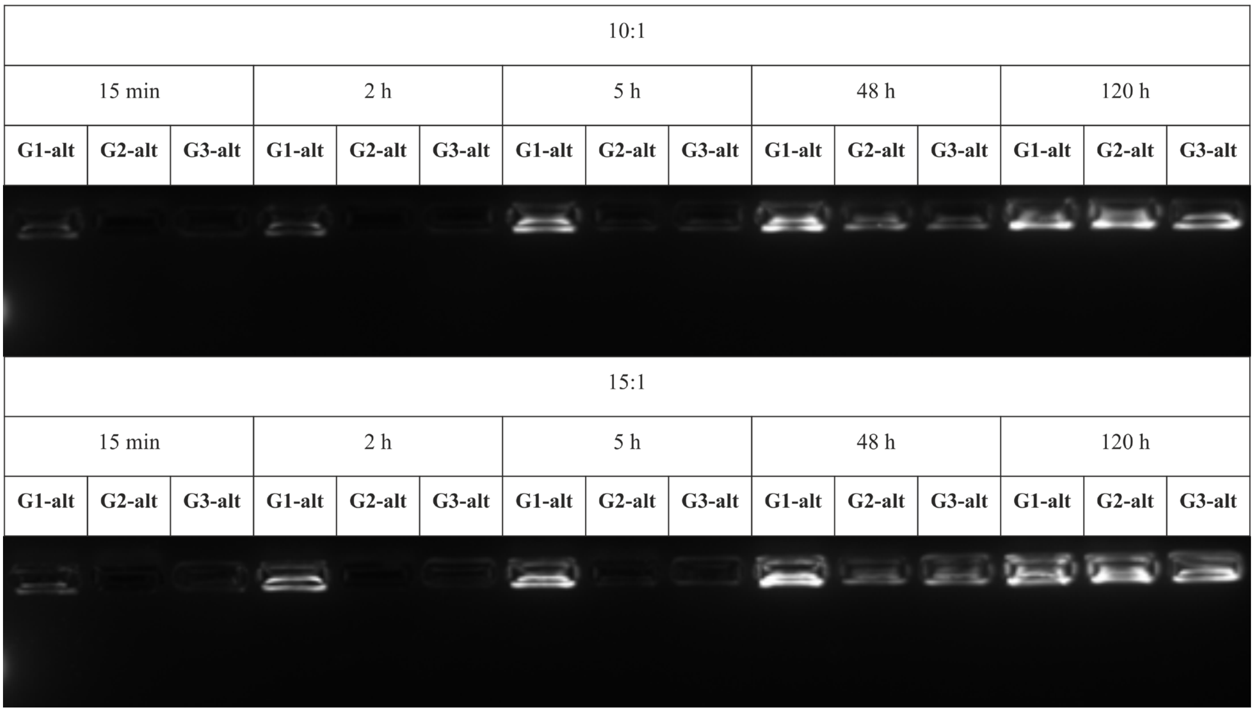

Figure S16. Temporal stability of the complexes. siBCL-2 concentration = 1.5 μM; TAE buffer, electrophoresis parameters: 30 min, 40 V. Incubation was carried out in the dark at room temperature (22 °C).

7. Evaluation of siRNA stability in the complexes with PAMAM-calix-dendrimers

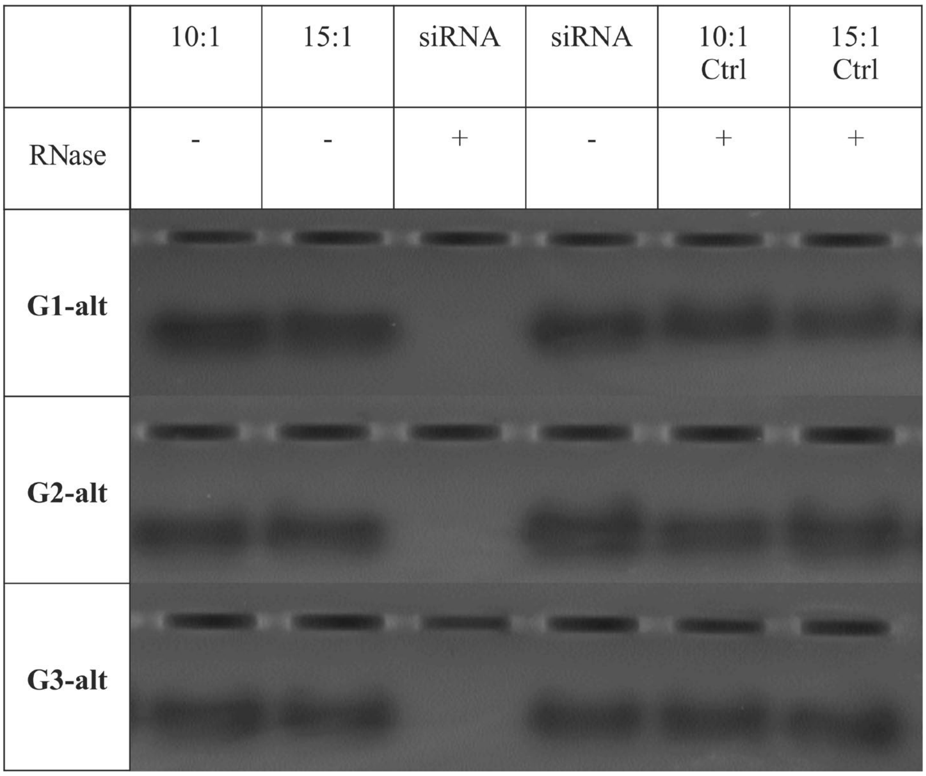

Figure S17. Evaluation of siRNA stability in the complexes with **PAMAM-calix-dendrimers** in the presence of RNases. The release of siRNA was carried out through competitive replacement of heparin; free siRNA was stained with ethidium bromide. siBCL-2 concentration = 1.5 μM; TAE buffer, electrophoresis parameters: 30 min, 40 V; incubation with RNases 30 min, 37 °C.

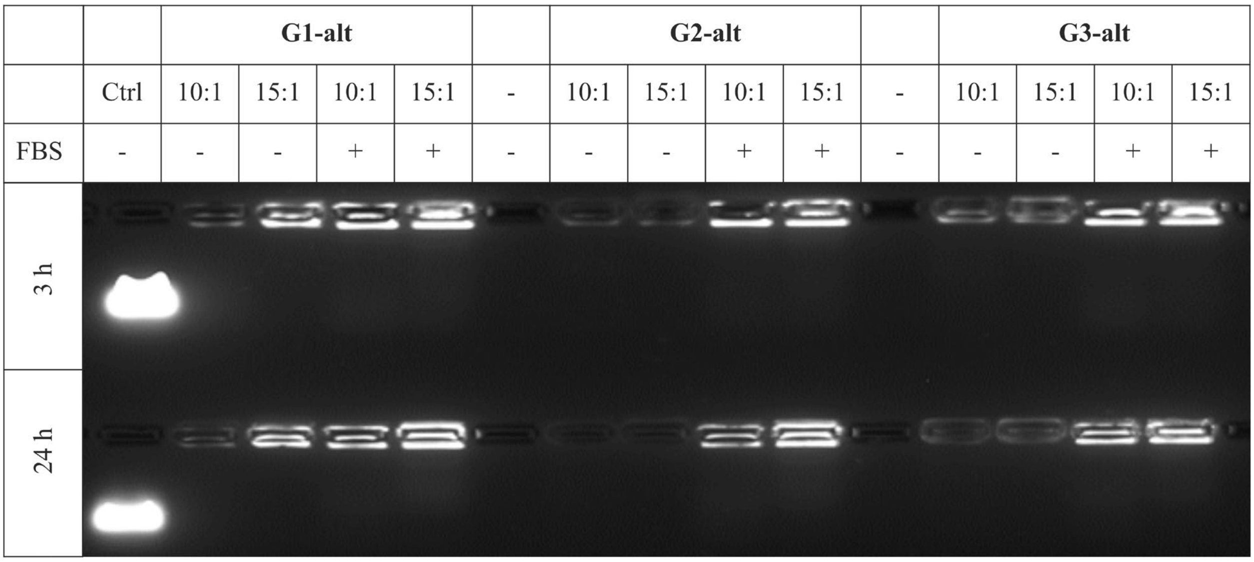

Figure S18. Evaluation of siRNA stability in the complexes with **PAMAM-calix-dendrimers** in the presence of 50% FBS. siBCL-2 concentration = 1.5 μM; TAE buffer, electrophoresis parameters: 30 min, 40 V; incubation with RNases 30 min, 37 °C.

8. Inhibition of PBMC viability by PAMAM-calix-dendrimers

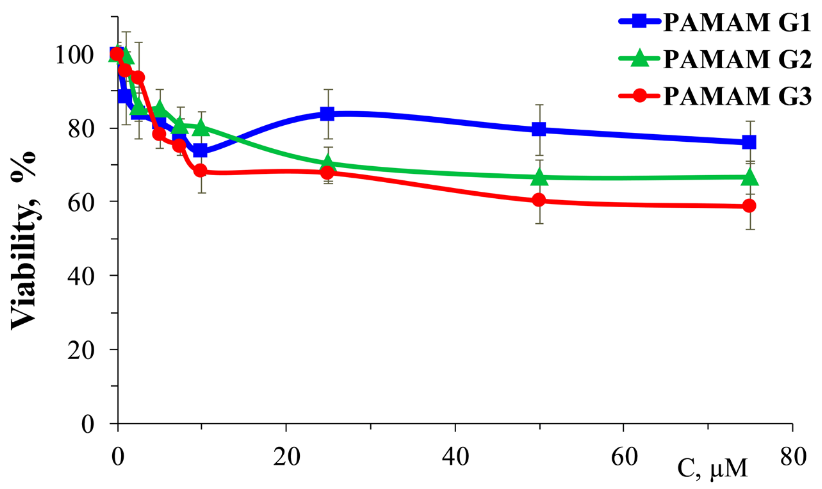

Figure S19. Inhibition of PBMC viability by PAMAM dendrimers after 72 h of treatment. Data are presented as percentage of viability of control cells, mean ± SD, n = 6.

9. Cellular uptake of PAMAM dendrimer/siRNA complexes

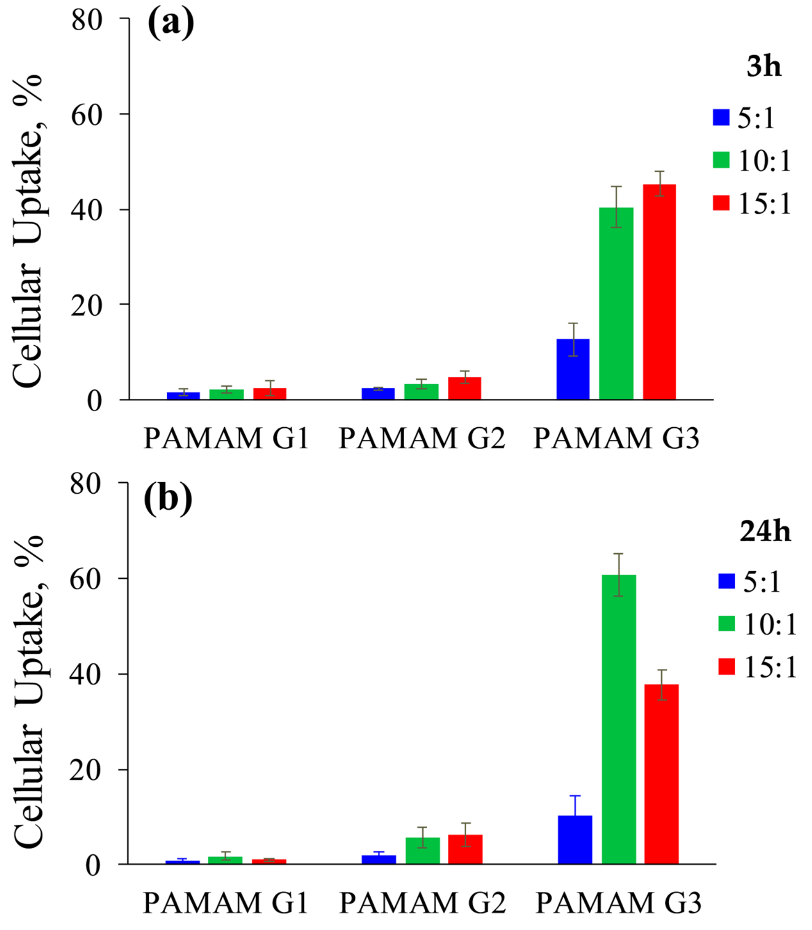

Figure S20. Cellular uptake of PAMAM dendrimer/siRNA complexes (ntRNA-FAM, 100 nM) at various N:P ratios in HeLa cells after (a) 3 h and (b) 24 h of incubation. Data were obtained based on fluorescence intensity from FAM-labeled RNA by flow cytometry. Data are presented as percentage of cellular uptake, mean ± SD, n = 6.

10. Fluorescent images of the internalized PAMAM-calix-dendrimer/siRNA complexes

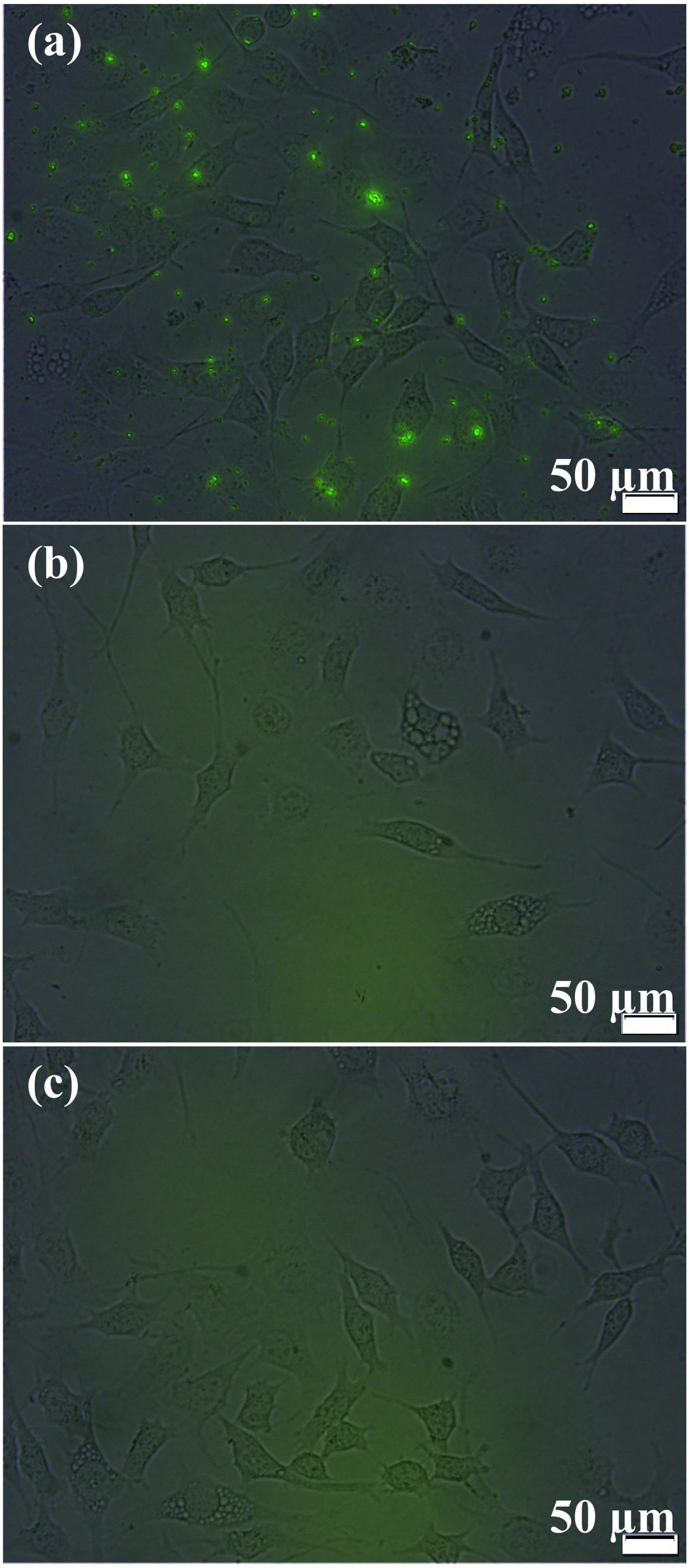

Figure S21. Superimposed fluorescent images of internalized the **PAMAM-calix-dendrimer**/ntRNA complexes (a) **G1-alt**, (b) **G2-alt**, (c) **G3-alt**. ntRNA concentration = 100 nM, incubation time 24 hours, HeLa cell line.

11. Dose-response curves of HeLa cells with PAMAM dendrimers

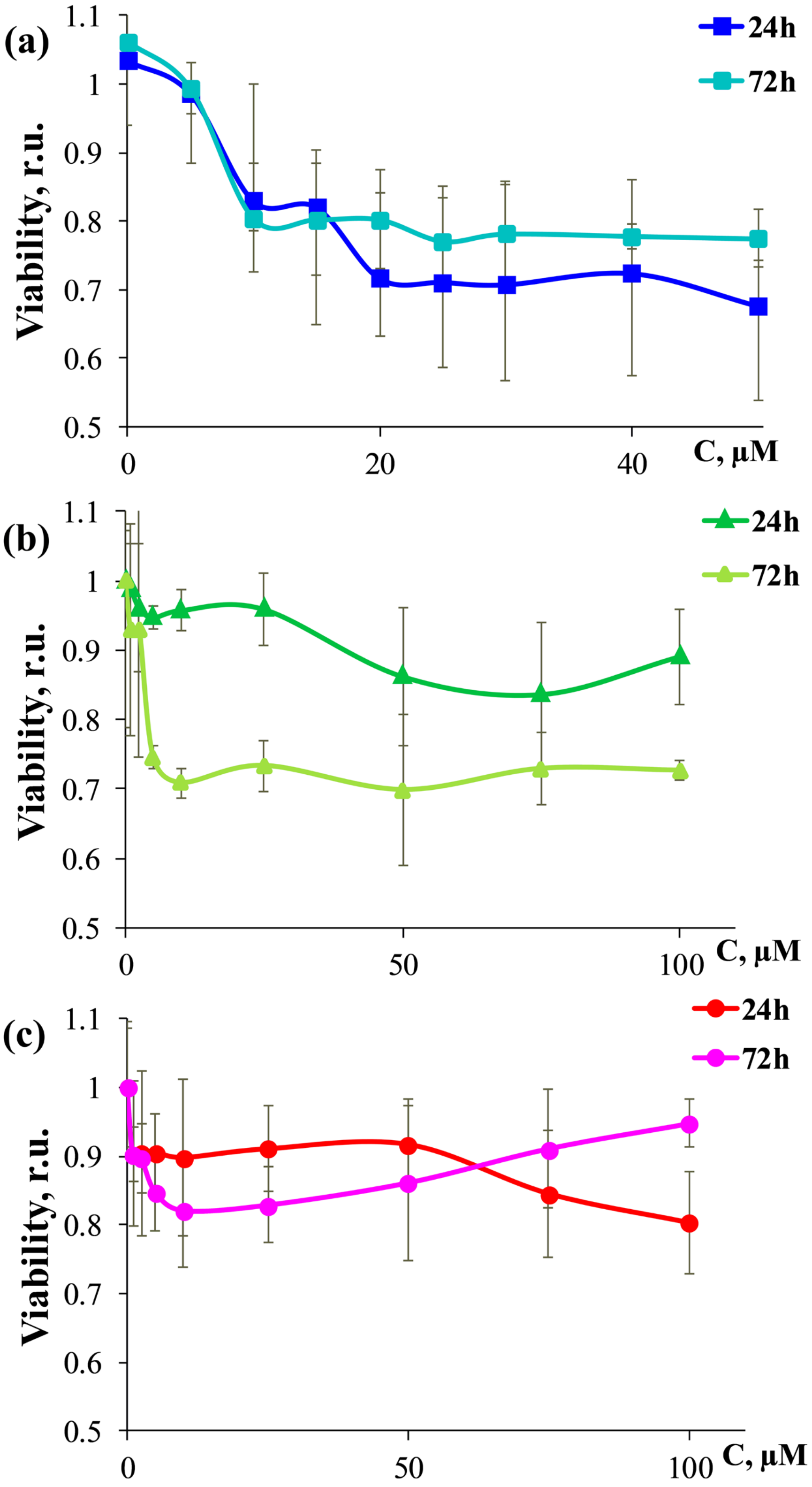

Figure S22. Dose-response curves of HeLa cells after 24 and 72 h incubation *per se* with (a) **PAMAM G1**; (b) **PAMAM G2**; (c) **PAMAM G3**. Data obtained from MTT assay normalized to control (untreated) cells. Data are presented as relative units (r.u.) of viability of control cells, mean ± SD, n = 6.
